# GRAFT: A Model for Evaluating Actuator Systems in Terms of Force Production

**DOI:** 10.3390/s20071894

**Published:** 2020-03-29

**Authors:** Hamza Baniata, Ahmad Sharieh, Sami Mahmood, Attila Kertesz

**Affiliations:** 1 Department of Software Engineering, University of Szeged, H-6720 Szeged, Hungary; keratt@inf.u-szeged.hu; 2 Department of Computer Science, The University of Jordan, Amman 11118, Jordan; sharieh@ju.edu.jo; 3 Physics Department, The University of Jordan, Amman 11118, Jordan; s.mahmood@ju.edu.jo; 4 Department of Physics and Astronomy, Michigan State University, East Lansing, MI 48824, USA

**Keywords:** IoT, GRAFT, Earth, graph theory, force, space-time

## Abstract

In the scope of evaluation methodologies for Internet of Things (IoT) systems, some approaches concern security, while others latency. However, some methodologies evaluate systems that contain active entities, so-called actuators. In this paper, we propose a novel methodology for evaluating such systems with actuator components using Graph Representation of the Angle of the Force and Time (GRAFT). GRAFT facilitates easy computation of the net force produced by physical or mechanical acts occurring on a daily basis on Earth. We use laws and definitions of physics describing the relations between Speed, Distance, and Time (SDT), apply them in a heliocentric system, and model the considered systems with a graph. The continuous movement of the Earth was shown to be weakening the total produced net force in some systems. We considered this weakening issue a problem, and we propose two possible solutions to overcome it by using restoration values, or reordering actuator sessions, in GRAFT to arrive to a more force-efficient system. We compared our default GRAFT algorithm to a special implementation using the Clock-Angle-Problem (CAP) for sessions. We also study and discuss an IoT-focused case for validating our approach, and we present a detailed explanation of the proposed GRAFT algorithm. The experimental results show the ability of GRAFT to provide highly accurate results, which also exemplifies that our GRAFT approach is programmable, hence deployable in real life scenarios.

## 1. Introduction

With 184 countries pledging to limit the increase in average global temperature to 1.5 °C above pre-industrial levels [1], and scientists recommending a decrease of up to 90% in greenhouse gas (GHG) emissions by 2050 [2], more ambitious pledges and further action is still required to meet those targets [3]. The European Commission has proposed a European Climate Law, which will turn political commitments into legal obligations for member states [4], referring to their 2018 commitment to reaching net-zero GHG emissions by 2050, calling for improvements in energy efficiency, which has been stagnating recently [5]. Following the mentioned insights, an evaluation model of actuator systems in terms of force exertion is helpful because it enhances the outcome of research and industries in terms of force efficiency, hence energy saving.

The Internet of Things (IoT) is a relatively new paradigm suggesting that things can be connected to the Internet and provide usable data about their environments. IoT as defined in [6] has emerged as a variety of technologies from Wireless Sensors Networks (WSN) to Radio Frequency Identification (RFID), that provide the capabilities to *sense*, *actuate* with, and *communicate* over the Internet. Consequently, things may be sensors [7], actuators [8], small processors [9], etc. that are able to communicate and process simple or complicated tasks. Some use cases of the IoT paradigm include smart homes [10], smart cities [11], smart vehicles [12], and smart health [13].

As clarified in [14], an actuator is a hardware component, which can act upon, control, or manipulate the physical environment, for example, by giving mechanical movement, optic, or acoustic signals. Actuators receive commands from their connected device, and translate electrical signals into some kind of physical action. Similar to sensors, actuators are typically connected to or are even integrated into a device, whereby the connection can be established by wires or wireless medium. If required, actuators can be configured using software but cannot run software themselves.

In some systems, geographically distributed sensors coupled with actuators are interconnected by wired/wireless networks to perform the entitled tasks, namely Wireless Sensor/Actuator Networks (WSANs) [15]. To gain more efficient management of sensors and actuators in an IoT system, we propose the evaluation of the energy consumed by the actuators in terms of force production. Actuators act according to the commands they receive from their connected devices, whether they were master devices or, in some systems, automatically react according to some sensed data by connected sensors. The mechanical configuration is usually provided by the creature of the system [16]. Examples of such systems might be fire handling systems, where sensors periodically report the status to the actuators in timely and reliable manner. Other examples such as safety-critical control systems and air conditioning systems include sensor and actuator entities too [15].

Many challenges are faced by IoT applications, such as security and energy efficiency [17], while evaluating such systems is usually provided in terms of security, data aggregation, response time, and network load. To evaluate the energy efficiency of such systems, electrical power consumption, total number of exchanged messages, or total up time of the system, are used. This research also suggests evaluating such systems in terms of energy consumption. However, since energy and force are strongly related physical concepts, energy consumed by the actuators in a day is evaluated in our approach by means of the force produced by the actuators.

**Figure 1:**
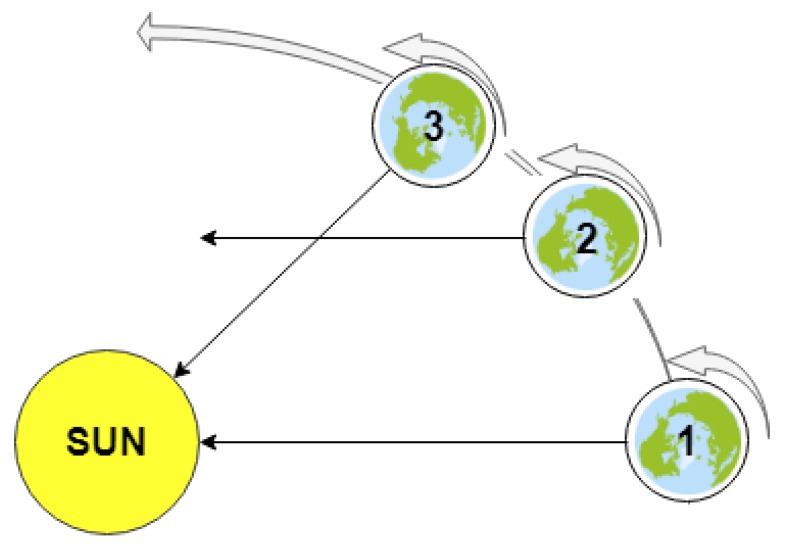
Stellar day vs. True Solar day: (**1**) time on Earth is exactly noon; (**2**) Earth completed a full rotation about its axis, but it is not noon yet due to the Earth revolution around the Sun; and (**3**) true noon of the following day.

It is well established that Earth is a heliocentric globe, which rotates around its own axis (by spinning) as it revolves around the Sun. The average rotational speed of a point at the equator on the surface of Earth due to its rotation about its own axis is about 465 m/s [18], which requires approximately 24 h to perform a full rotation determining the time through a day. The direction of Earth spinning is from west to east (counter-clockwise as viewed from North Star or Polestar Polaris). On the other hand, Earth revolves around the sun in an elliptical orbit with an average speed of almost 29.8 km/s [18], which requires approximately 365 days to complete a full revolution. Accordingly, the superposition of Earth movements may result in relatively small changes of the observed position of the Sun in the sky, as demonstrated by Figure 1.

Relative to the Sun, the Earth’s rotation period (true noon to true noon) is its true solar day [19], which is different from the stellar day, as shown in Figure 1. Earth’s orbital motion, the eccentricity, and inclination of Earth’s orbit, however, vary over thousands of years, so the annual variation of the true solar day also varies. It is obvious that, on a planet such as Earth, the stellar day is shorter than the solar day [19].

**Figure 2:**
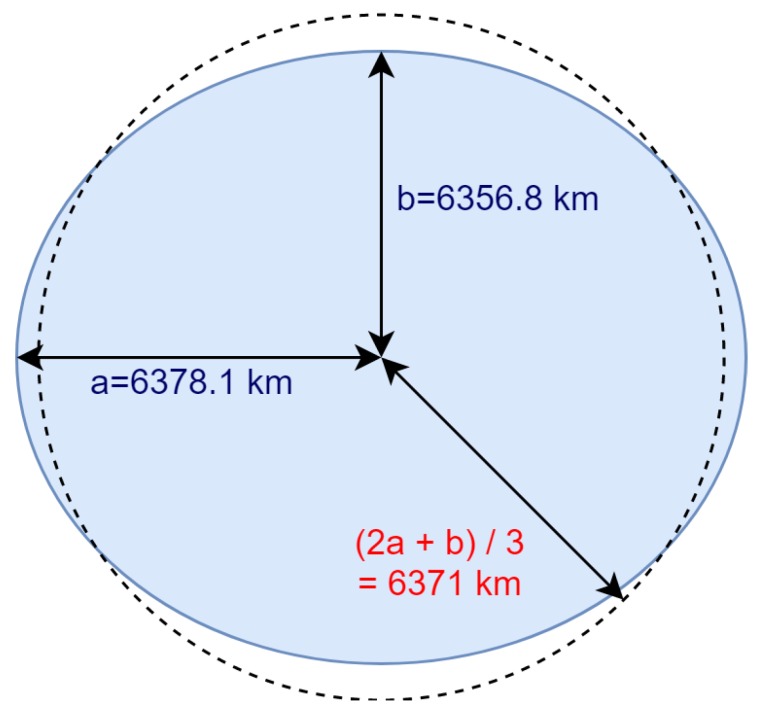
Radius of Earth: (**a**) radius from the center of Earth to a point on the equator; and (**b**) radius from the center of Earth to the pole. Average radius of Earth: $\frac{2a+b}{3}$.

**Figure 3:**
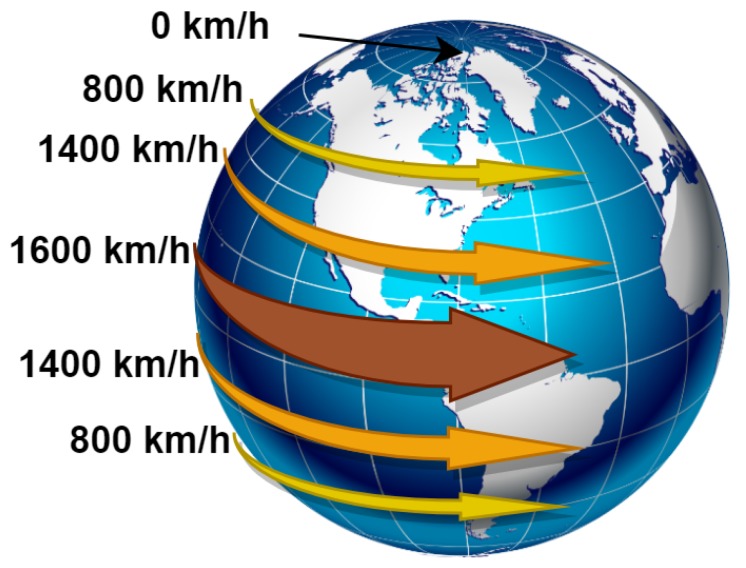
Rotational speed of Earth differs according to the latitude line. In Canada, it equals 800 km/h, while it doubles in Colombia to be 1600 km/h.

According to the Heliocentric theory, there are different measurements—since it is not a perfect sphere—for the radius of Earth from its center to the surface [20]. There are also different measurements of the rotation speed of Earth [21] due to different latitudes. Figure 2 and Figure 3 clarify these facts.

**Figure 4:**
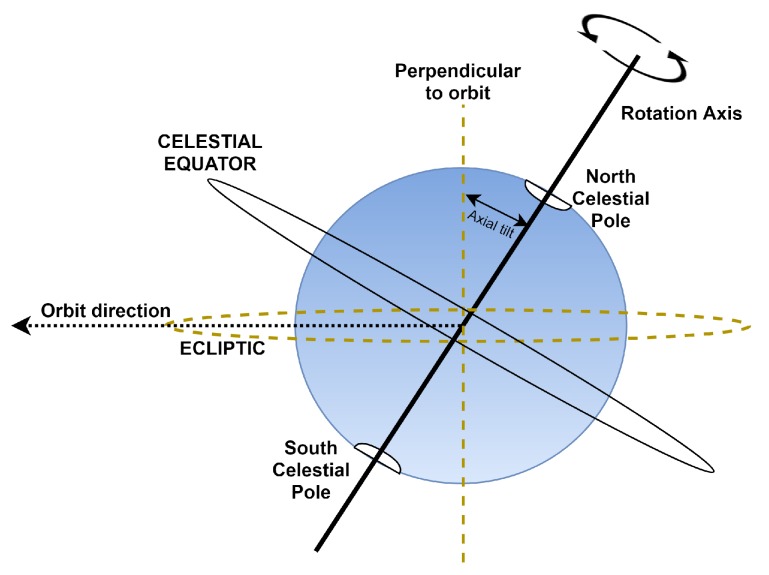
Rotational axis, perpendicular- to-orbit axis, and axial tilt.

As shown in Figure 4, The rotational axis is the imaginary line connecting North Pole, South Pole, and center of Earth. The perpendicular axis is the imaginary line that is perpendicular to the imaginary orbital path that Earth walks around Sun. the axial tilt is the angle between those two imaginary lines, which equals, approximately, 23.5°. Although we believe the mentioned pieces of information are sufficient to clarify our proposal concepts, we direct the reader to investigate them deeply, if needed, at [18,22,23,24].

We use notations from graph theory to model IoT systems. A graph ‘*G*’ is an ordered pair (V(G), E(G)) consisting of a nonempty set V(G) of vertices (or nodes), and a set E(G), disjoint from V(G), of edges. If ‘*e*’ is an edge, and ‘*u*’ and ‘*t*’ are vertices such that ‘*e*’ connects ‘*u*’ and ‘*t*’, then *e* is said to join *u* and *t*, while the vertices ‘*u*’ and ‘*t*’ are called the ends of ‘*e*’. A graph with no loops and no multiple edges is called a simple graph. According to whether each edge has an assigned orientation, graphs can be classified into directed or undirected [25].

With each edge ‘*e*’ of ‘*G*’, let there be associated a real number w(e), called its weight. Then, ‘*G*’, together with these weights on its edges, is called a weighted graph. Weighted graphs occur frequently in applications of graph theory. In the friendship graph, for example, weights might indicate intensity of friendship; in the communications graph, they could represent the construction or maintenance costs of the various communication links. The centrality concept in graphs, which is the study of statistical distributions of various quantities/attributes co-related with the nodes of a graph, describes the aggregate properties of the many elements that compose that graph [26]. However, for more detailed information on graph theory concepts, we direct the reader to seek them in [25,26,27].

The ability or capacity to do work (work = force × distance) is called energy. It was shown by Jansen and Stevels [28] how each physical act consumes and produces energy at the same time. Energy produced by humans or machines could be electrical as communications between brain neurons, or thermal by muscular work [28]. Energy can also be light, produced by some machines or creatures. Other forms of energy are, for example, of gravitational, acoustic, or magnetic origin. The energy is expressed in units of “Joule” in the SI system of units [29].

Energy was also proven to be convertible from one form to another, but can be neither destroyed nor created, which is called The Universal Law of Energy Conservation [29]. This law affirms that the total amount of energy (including mass as a form of energy) in the universe is fixed. Energy is defined in the Cambridge Dictionary as “The power from something such as electricity or oil that can do work, such as providing light and heat”. Energy as defined in physics is “An exertion of power” [30]. Here, we see that work or energy is directly related to the force of interaction, be it electrical, gravitational, magnetic, etc. As a definition, Force is “the strength or energy as an attribute of physical action or movement” [31]. Force is represented by a vector that defines the direction of the enforced energy and its magnitude. Vector components are usually perpendicular to each other, although they can also be in a parallelogram configuration. For more information on the topic, we direct the reader to investigate force concepts in [32].

In light of the definitions discussed above, we aim to propose our novel idea for evaluating the efficiency of actuators that are deployed within certain given conditions in some IoT environment. The purpose and main concern of our proposal is to compute the total net force, which can give an indication of how efficient the evaluated system is. In this paper, we propose the GRAFT (Graph Representation using the Angle of the Force and Time) model, which can be used even on different planets of our solar system, as well as for other solar systems that have planets moving around a star. Our work also includes a proposed algorithm with variables that can be modified for different actions, locations, planets, or solar systems.

When applying our proposed evaluation model to a given actuator system, the result presents how weakened the produced force can be, due to the continuous movement of the Earth. The proposed evaluation methodology can also help deploy a more efficient system using the available resources, in terms of net force produced. That is, a system ‘A’ that consumes less energy than system ‘B’ to exert the same value of total produced force, or ‘A’ that consumes the same amount of energy consumed by ‘B’ to exert more force, is the efficiency we are seeking.

The following sections of this paper are as follows. Section 2 represents the state of the art regarding evaluation methodologies for actuators systems. Section 3 presents the foundations of the GRAFT model, its definitions, conditions, parameters, and assumptions. In Section 4, a case study is proposed, the GRAFT algorithm is presented, into which the proposed case was applied and explained in detail, and then the results of the application are provided and discussed. For the sake of validating our algorithm, we compare our results to the results we obtained using a different mathematical approach, namely the Clock-Angle-Problem. Finally, Section 5 concludes our work.

## 2. State of the Art of Evaluation Methodologies in IoT Systems

In the field of IoT systems with actuators deployability, previous and current research articles have evaluated their proposed systems using methodologies that mainly targeted performance issues such as latency, power consumption, and resource allocation. The authors of [33] analyzed the performance of IEEE 802.11ah technology, in an actuation use case for connected lighting, from the perspective of latency and power consumption. The authors of [34] proposed two architectural approaches for smart building systems, which may include actuators connected to the network. The two approaches were compared with each other and evaluated in terms of memory allocation, energy consumption, and latency per transaction. The authors of [35] compared open standards that are commonly used in IoT systems with a probability of actuators existence. Their evaluation metrics included storage occupation, memory usage, and response latency. The authors of [36] investigated a mobile, wireless sensor/actuator network application for use in the cattle breeding industry, and evaluated the performance of their design by comparing simulations to field experiment considering different metrics such as delivery rate in realistic situations and the aggressive behaviors of bulls. While Malik et al. [8] and Palacios and Córdova [37] evaluated their proposed system using a case study, the case study in [8] was validated for connection and response times of system entities through the experiment. The KASEM data visualization tool proposed in [38] was evaluated on the basis of machine load, IoT platforms load, and virtual users’ number.

There are hundreds, perhaps thousands, of papers, surveys, and proposed ideas discussing a very wide range of issues relating to Earth, astronomy, and IoT knowledge [39,40,41,42]. However, as we searched extensively for related works to ours, we found that no previous work discussed the computation of net force produced by actuators on Earth conceptually as described in this paper. To the best of our knowledge, this paper proposes a novel model that no researcher before presented the same way we do.

## 3. Foundations of the GRAFT Model

First, the actuator scenario that can be applied into the GRAFT model goes as the following:

1. Several tasks need to be performed by an actuator each day, in different times (named *sessions*), through the day.
2. Each task has to be performed within its predefined session through the day.
3. A session has a beginning time and an ending time. Between the beginning and the ending time of a session, the task (with all its cycles), has to be performed.
4. Each task performed by an actuator has several working cycles. The number of working cycles in a task might be similar to, or different from, the number of cycles performed in other tasks. However, a cycle in a task is similar to all other cycles in the same or other tasks. A working cycle has a predefined known amount of produced force.
5. The cycles of a task may be performed in any time within the session.
6. The number of tasks, the order of the tasks, and the number of cycles performed in each task, is a daily routine that will consume/produce the same amount of energy, hence the same amount of daily force.
7. The GRAFT model computes the total net produced force, in a day, by this actuator, performing the predefined tasks in the predefined sessions.

Accordingly, a graph ‘*G*’ is built where:

1. The nodes of the graph represent the sessions where tasks are performed by an actuator, producing similar type of force.
2. One extra central node is added to the graph and an edge is defined between this extra node and all other nodes. This should always produce a graph with the following properties:
  - simple;
  - two-leveled acyclic tree;
  - complete bipartite;
  - planar; and
  - has one face.
3. The weight of each edge equals the ratio value of cycles performed in the adjacent node, to the total number of cycles performed in a day.
4. The positioning of each node is then determined according to the starting time of the session and the session duration. The position of each session is stable relative to Sun position in the sky and not affected by Earth rotation about its axis, because the position of the sun for an observer standing on Earth defines the time for that person. For example, if an actuator is supposed to perform a task during the time 12:00–13:00, it means that this actuator will enter the session/node at 12:00, and will have 60 min to perform the task. That is, the movement of Earth for this static actuator is causing it to enter/exit the session.

The model is studied on the planet Earth, but can be—by changing some values in its algorithm—generalized to other similar-to-Earth planets.

### 3.1. Definitions

- A node represents a session. A session is a vertical sector that lies between two lines drawn on the surface of Earth. A sector has two endings at north and south, where the two lines meet. The first line represents the starting time of a session, while the second line represents the ending time of the session.
- A task is all the predefined number of working cycles that should be performed in a named session.
- A cycle is a predefined unit of tasks. A task is at least one cycle.
- Session time is the number of minutes during which the actuator has to perform the task.
- Travelled distance is the distance the actuator travels from the entering point in a session, until the exiting point out of a session.
- Angle of the session is the angle between two lines: the first is the line connecting the central node to the entering point of a session, and the second line is the one connecting the central node to the exiting point of a session.
- Direction of the session is the estimate bearing of the line connecting the central node to a point between the entering point and exit point, relative to the *x*-axis.
- The central node represents the point in the center of Earth where the speed of rotation equals to zero.

As previously mentioned, the weight ‘*W*’ of an edge evaluates the ratio of the number of cycles ‘c’ performed in the adjacent session to the total number of cycles ‘*T*’ that should be performed by all tasks in a day. However, R equals ‘1’ cycle, hence (W) can be calculated using Equation (1).

(1) $$W=\frac{c*R}{T}$$

The force ‘*A*’ produced by a task that performs ‘c’ cycles equals A(c) (i.e., the force produced by one cycle A(R) multiplied by c). Consequently, as presented in Equation (2), the ratio of the produced force by a performed task A(c) to the expected produced force by all tasks performed in a day A(T) equals *W* too.

(2) $$W=\frac{c*A(R)}{A(T)}$$

All nodes in the GRAFT are stable angle wise. This means that, since calculating the net produced daily force depends on the direction (its sine and cosine), as will be shown, it matters if the angle between any two edges differs. In addition, despite maintaining the graph properties of being complete bipartite, acyclic tree, and planar with only one face, for *any* other similar case, the graph will have different numbers of nodes, edges, weights, and angles for each case. Hence, an algorithm is needed to compute the daily produced force in any similar system. The direction of each edge is considered the vector of the force, which is produced by the task and performed in the adjacent session. The weight of that edge is considered the magnitude of that force.

### 3.2. Conditions

- The proposed model is based on the true solar day concept.
- The average rotation speed of the surface of Earth and the average radius of Earth are used in the proposed algorithm.
- As there are various different parameters used in the GRAFT model and algorithm, it is more suitable to present them in a tabular form. Table 1 presents those parameters.

### 3.3. Assumptions

- If all tasks were performed with all their cycles at the same session, then this would produce 100% force, because the direction of force produced by all performed cycles is the same, and the weight of the edge representing the session equals exactly 1 (i.e., the degree of force weakening is zero).
- Actuator ‘*M*’ that performs ‘*c*’ working cycles a day is better than actuator ‘*N*’ that performs the same number of cycles if *M*’s total net daily produced force is higher than *N*’s (for example, A(M) = 80% while A(N) = 75%). The reasons for the difference are locations of actuators, session times, or both.
- Edges of the graph indicate the direction of nodes from the center. This is done to make it easier to picture the idea of the mathematical trigonometric calculations of the net produced force. However, it is not important to consider whether the edges’ direction goes out of the source node to the termination nodes or in the opposite direction, because the net force will remain the same in both cases.
- The source node in GRAFT lies in the center of Earth and *not* on the North Pole. In fact, if the source node were thought of as located on the surface of the Earth, then its position would differ every second as Earth rotates about its axis.

## 4. The GRAFT Algorithm and a Case Study Application

It would be easier to deliver the idea of our proposed evaluation methodology using a case study. In this section, the net magnetic force produced by a given machine that works five times a day is considered as a case study. In this section, we do the following:

1. We introduce the suggested case study.
2. We apply the GRAFT algorithm on the suggested case study, and explain each step of the proposed algorithm.
3. We calculate the resultant daily net force produced.
4. We validate our method by comparing its results with the results obtained using the Clock-Angle-Problem formula.

### 4.1. The Suggested Case

**Figure 5:**
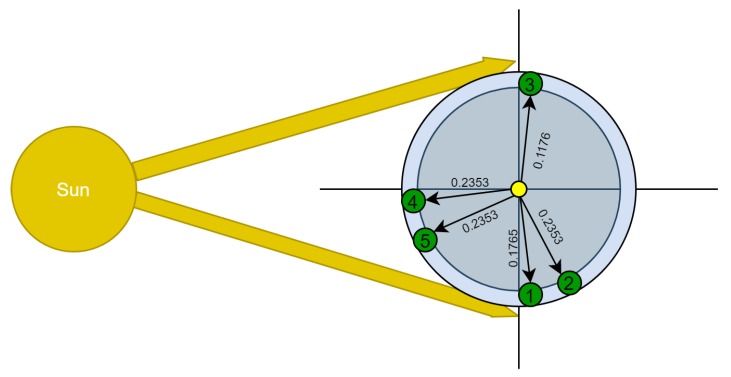
GRAFT model applied on the studied case. Green nodes (1–5) represent the first to fifth sessions, respectively. The yellow node represents the central node. Weights of edges represent the ratio of adjacent session’s cycles to the total daily cycles.

A machine works five times a day. Each working task has pre-defined number of working cycles that should be performed with a total of 17 working cycles per day. Each cycle takes exactly 1 min, and produces certain known amount of force. The time when each task should be performed is pre-defined and called a session. The time for performing each task is less than the session time; thus, the machine has plenty of time to do its job. The directed simple graph in Figure 5 represents the five sessions with their topological order through one day, as viewed by an observer whose sight line is perpendicular to the the imaginary orbital path (Earth turns counter-clockwise). In our proposed case study, W(1) is approximated by 0.0588, because its ratio is 1/17. Consequently, the ratios and the weights of first, second, third, fourth, and fifth tasks are approximated by the values presented in Table 2. For example, the third task consists of two out of seventeen cycles, which is 2/17 relative to total performed cycles through one day. Hence, W[3] = 0.1176.

In the first session, three cycles should be done; this session starts at 18:00, and extends for 90 min. In the second session, four cycles should be done within the following 90 min of the end of the first session. That is, it starts at 19:30 and ends at 21:00. In the third session, two cycles should be performed within a 70-min session starting at 04:50 and ending at 06:00. In the fourth session, four cycles should be performed. The fourth session starts exactly at 12:00 (noon), and extends for 191 min, hence ends at 15:11. In the fifth and final session of the working day, another four cycles should be done within the following 120 min of the fourth session. Hence, the fifth session starts at 15:11 and ends at 17:11. If this routine is daily committed to, the net force calculated next would be the daily produced net force by that machine.

Since the rotation axis of Earth is not perpendicular to its orbit about the sun, as shown in Figure 4, it must be clear that the polar plane drawn in Figure 5 is not in the celestial equator plane but in the ecliptic level.

### 4.2. Application

In this subsection, we provide a step-by-step explanation of the proposed GRAFT algorithm. For this reason, we give each major step in Algorithm 1 a number that indicates that step. Furthermore, We apply the calculations of the algorithm to the previously proposed case, leading to computing the net force. Our methodology of computing the net force will be conducted as in the following description:

The user inputs the number of sessions, which must be bigger than zero. The user then inputs the number of cycles that should be performed in each session. After that, the algorithm draws the graph and defines each edge’s weight. The user then inputs the starting and ending times of each session. The algorithm then calculates the session time by subtracting the start time from the ending time of the session.
**Algorithm 1:** The GRAFT algorithm.
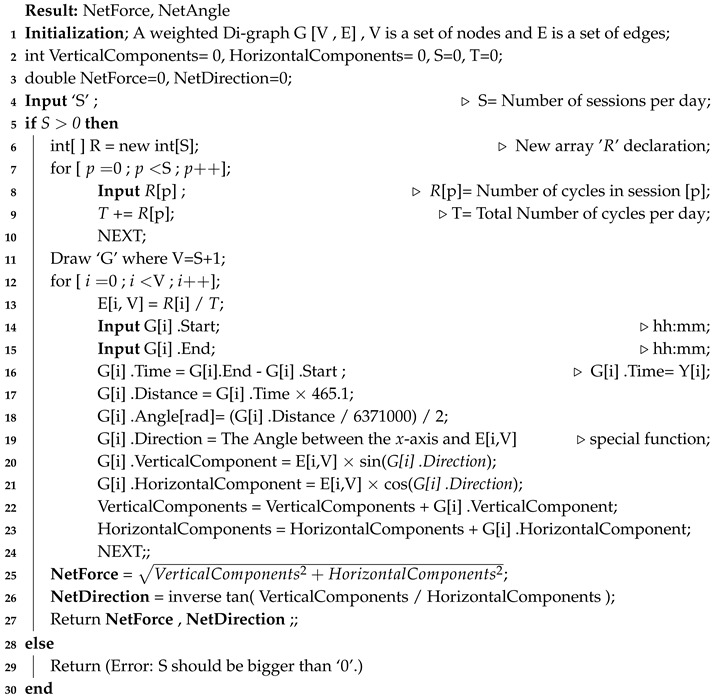


**Figure 6:**
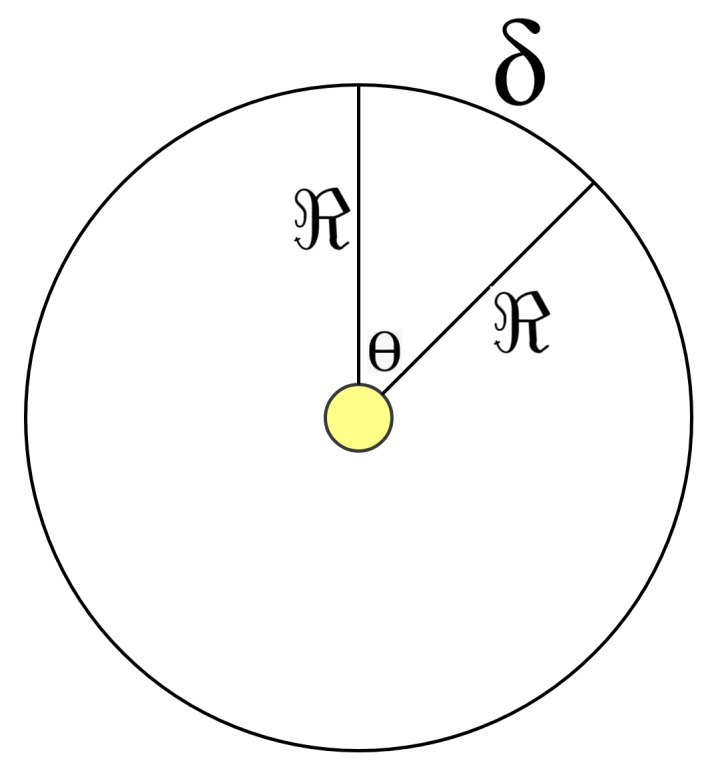
In any circle, the relation between the length of Arc ‘δ’, the radius ‘*ℜ*’, and the angle ‘Θ’, measured in radian system, is δ = Θ×ℜ.

After that, it calculates the distance that the actuator travels while it is in the session time (using the average speed of Earth). Then, it calculates the angle of the session drawn on the central node (using the average diameter of the planet). Consequently, the algorithm finds the direction of the session relatively to the *x*-axis. This latter value is used then as the direction of the force vector, which is used in computing the vertical and horizontal components of it. At the end of the algorithm, the net force produced by the system is computed. Each step of the algorithm is numbered and explained in detail as the following:

- (1)–(3): Initialize the variables of the algorithm.
- (4): User inputs the number of sessions in a day ‘S’.
- (5): The algorithm checks if the number of sessions ‘S’ equals one or more, otherwise the calculations will be meaningless.
- (6): Declare an array that will hold the values of cycles per session.
- (7)–(10): Start a for loop in which the user inputs the number of cycles per session, and consequently the algorithm computes the total number of cycles per day.
- (11): Draw the graph according to the number of sessions.
- (12)–(21): Start a for loop in which each session’s weight, vertical component, and horizontal component are calculated.
  – (13): Compute the weight of the session using Equation (1).
  – (14) and (15): User inputs the start and end times of the session.
  – (16): The algorithm computes the total time of the session accordingly.
  – (17): The algorithm computes the session’s distance. If both the rotational speed at a given point on the surface of Earth and the movement time between two points are known (computed in Step (16)), then the distance ‘δ’ traveled can be calculated from the relation of speed–distance–time. This relation defines the distance as the speed of movement multiplied by the time [43], as given in Equation (3). This means that an observed object that moves with speed ‘ϑ’ will pass the distance ‘δ’ in time of travel ‘Y’.
    (3) δ=ϑ×Y
    The algorithm calculates each session’s distance ‘δ’. Session’s distance represents the traveling distance for an entity located on the surface of Earth, from the starting point of a session (west) to the ending point of the session (east). For the current example, the results of this step are shown in Column 4 of Table 3.
  – (18): Use the result of Step (17) to calculate the angle ‘Θ’ in radians measured between two lines. The starting line of Θ is the one connecting the central node to the starting point of session, and the ending line of Θ is the one connecting the central node to the ending point of the same session. Angle Θ is calculated according to Equation (4), which is clarified in Figure 6.
    (4) $$\Theta =\frac{\delta }{ℜ}$$
    The results of this step are provided in Column 6 of Table 3. The dividing-by-2 part in this step is done for partitioning the angle in half. That is, the direction typically appears between two lines; hence, one line is needed to represent the direction of the session (node) relative to the *x*-axis. The results of this are provided in Column 7 of Table 3.
  – (19): In this step, compute the direction ‘Λ’ of the found edge in Step (18) relative to the *x*-axis. To do so, perform the following:
    * For the first session, the starting time is 18:00, hence the direction of the starting time relative to the *x*-axis is 270°. Consequently, the direction of the session equals 270 plus ($\Theta_{1}/2$).
    * For the second session, the direction equals the ending time direction of the first session (which is 270 + $\Theta_{1}$) plus ($\Theta_{2}/2$).
    * For the third session, the ending time is 06:00, whose direction relative to the *x*-axis is 90°. Hence, the direction of the third session equals the direction of the ending time minus ($\Theta_{3}/2$).
    * For the fourth session, the starting time is 12:00, hence the direction of the starting time equals 180°. Consequently, the direction of the session relative to the *x*-axis equals 180 + ($\Theta_{4}/2$).
    * For the fifth session, the direction of the session equals the direction of the ending time of the fourth session (which is 180 + $\Theta_{4}$) plus ($\Theta_{5}/2$).
    The results of this step are shown in Column 8 of Table 3. The values in the table are represented in degrees, but the computations were actually done using the radian system. The approximate bearing of the edges are shown in Figure 5.
  (20) and (21): The algorithm in this step computes the vertical and the horizontal components of the edge, respectively, as a vector, and uses the weight of the edge as its magnitude. Equations (5) and (6) are used for that, and Table 4 presents the results.
    (5) VerticalComponent=W×sin(Λ)
    (6) HorizontalComponent=W×cos(Λ)
- (22)–(24): The algorithm in this step updates the values of the public variables “VerticalComponents” and “HorizontalComponents”, respectively, and moves to the next session.
- (25)–(27): Once the FOR sentence terminates, all needed values to find the total net produced force for all sessions become available. Using those values, the Pythagorean theorem, and classical trigonometric calculations, the algorithm computes the net force and its direction. Finally, the algorithm returns the public values NetForce and NetAngle in Step (27).

### 4.3. Results

**Figure 7:**
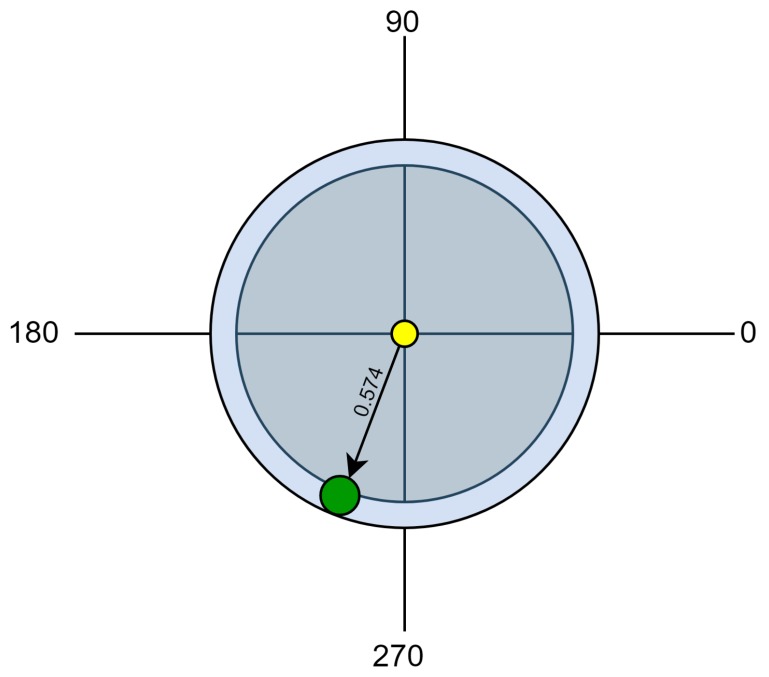
The direction and the magnitude of the resultant net force vector.

For the proposed case, the computed net force equals approximately 57.4% out of the optimal 100%. This means that GRAFT model could represent how the movement of Earth weakened the produced force with a percentage of 42.6%. Moreover, the computed direction of the net force vector relatively to the *x*-axis is approximated by 238.45°. Figure 7 presents the mentioned results.

According to the assumptions of the GRAFT model, a system, in which a machine works 17 cycles, that produces daily net force more than 57.4%, is better than the one studied in our proposed case. The GRAFT model, along with its algorithm, was applied to calculate the total net force produced by a machine performing seventeen cycles of work within pre-defined time sessions. The application of GRAFT on the mentioned case showed that the ratio of the net force to the optimal 100% ratio maps to a number between 0 and 1. If the 100% ratio is required in some situation, a **restoration value** is needed to gain back the weakening of force caused by the rotation of Earth. A restoration value might be one or several cycles done within pre-defined time sessions. For the correctness of a proposed restoration value to be tested, the GRAFT model must be applied one more time for combining the original net force vector with the proposed restoration value vector. Consequently, if a final vector whose magnitude equals to precise ‘1’ is gained, the proposed restoration value is successful.

### 4.4. Validation

In this subsection, we compare the sessions’ directions we obtained when applying the GRAFT model—which were computed on Line 19 of the GRAFT algorithm, and provided previously in Table 3—with the sessions’ directions we obtained by applying the mathematical Clock-Angle-Problem (CAP) formula in our algorithm. To do so, we implemented the GRAFT algorithm using Python 3.8 (https://github.com/HamzaBaniata/GRAFT) and compared the results of GRAFT with the results of the CAP formula.

**Figure 8:**
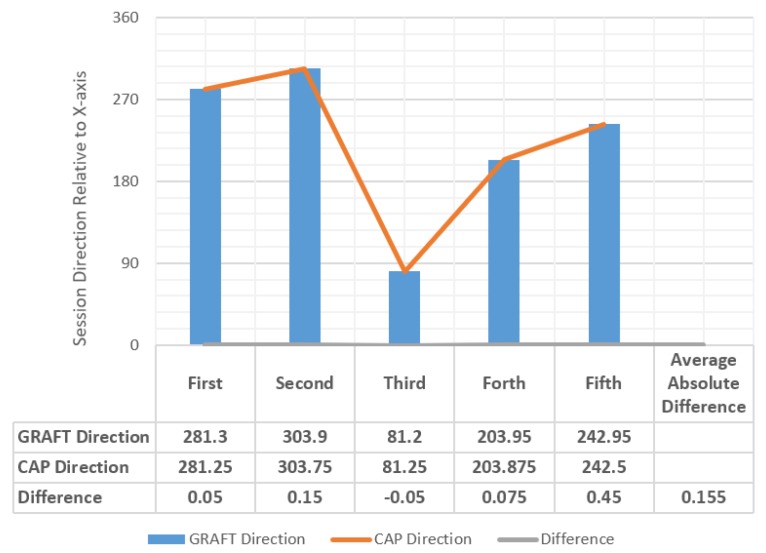
Comparison results of GRAFT and CAP.

**Figure 9:**
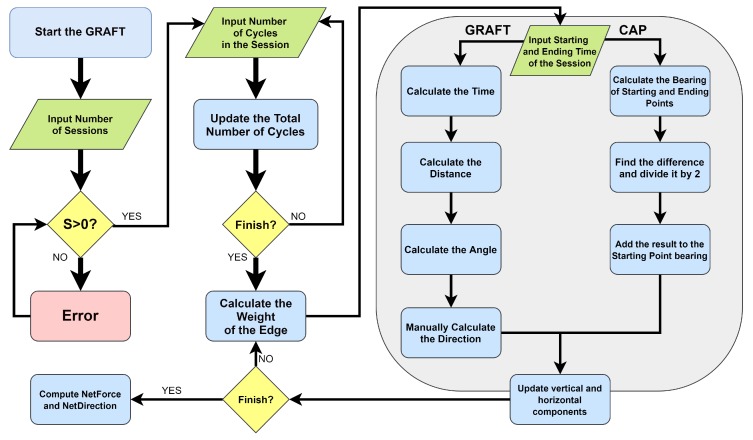
The FlowChart of the GRAFT model including the place of the Clock-Angle-Problem formula.

The CAP formula calculates the angle between the beginning of the session and the end of the session Θ using Equation (7). The CAP formula is also capable of computing the direction according to the time (input in 12-h type), as shown in Equation (8). However, since we suggest that time is input in 24-h type, we divided the value of Λ by 2, to cope up with this, as in Equation (9). Algorithm 2 presents the algorithm we used in our implementation, and Equation (9) presents the mathematical approach of direction solution. The chart and the companion table in Figure 8 shows the results we gained of the validation, while Figure 9 shows where exactly GRAFT calculations differed from CAP calculations. The case study we presented above was intended to show how we could compute the angles and the directions observably. The validation and our implementation, on the other hand, show that the GRAFT algorithm and its results can be implemented in a computerized application using the CAP formula, which can provide identical results to the original algorithm, if the case study included a spherical or close to spherical shaped planet.
**Algorithm 2:** Clock-Angle-Problem.
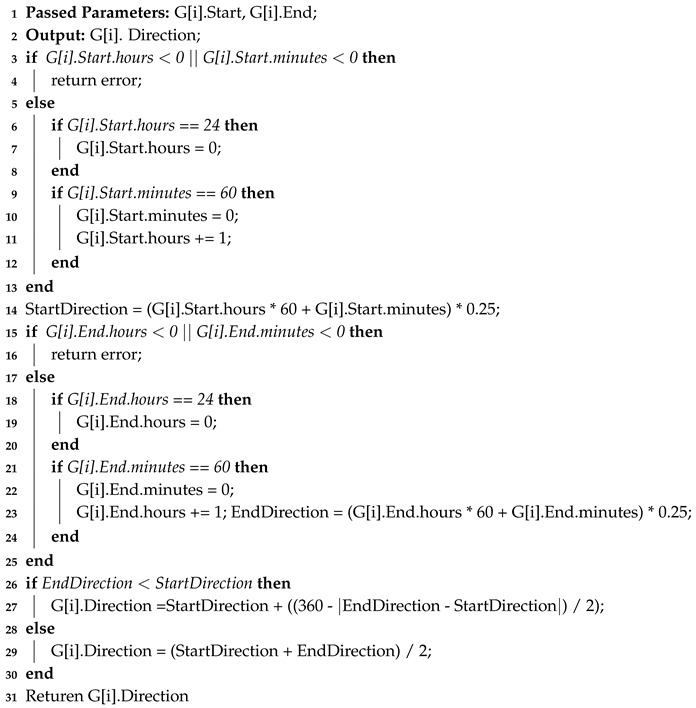


In contrast, our results were more accurate since GRAFT considered the fact that Earth is not a perfect sphere. The GRAFT results were quite close to the theoretical results obtained from CAP with an average absolute difference of 0.155° in the direction computing. This indicates the correctness of our proposed methodology in computing the angles and the direction of sessions.

(7) Θ=|Λend−Λstart|

(8) Λ=0.5*(60*H+M)

(9) $$\Lambda =\frac{0.5*(60*H+M)}{2}$$

where Λ is the direction; *H* is the hour; and *M* is the minutes past the hour.

### 4.5. Discussion

We suggested a case in Section 4.1 wherein one actuator performs 17 cycles of work in five different times (sessions) each day. We represented the case using the graph theory concepts of nodes, edges, weights, and directions. Then, as shown in Section 4.2, we applied our proposed GRAFT algorithm on the case, which, sequentially, drew the graph and computed the weights of edges, the time of each session, the travelled distance in the session, the angle of each session, and the direction of each session. Accordingly, GRAFT could compute the vertical and horizontal components of the force vectors, and output the net force and net direction. The results of the application are presented in Section 4.3.

Although our approach appears to be replaceable by a simple vector addition, in reality, it is not as simple as it seems. The simple vector addition is applicable in the case where the planet is a perfect sphere. However, the results will be different when the planet is not a perfect sphere. This is demonstrated by the comparison presented in Section 4.4. The comparison of CAP and GRAFT results showed a small difference in the measurement of session directions. That is, the GRAFT model provides solutions for problems CAP cannot solve; CAP solves only a special case of problems solved by GRAFT, where the planet is a perfect sphere. Comparing the two models, we arrive at the following conclusions:

1. The CAP formula and the GRAFT algorithm provide exactly the same results if and only if the system is deployed on a perfectly spherical planet.
2. If the planet deviates from spherical symmetry, GRAFT gives more accurate and slightly different results compared with the results of CAP formula. Accordingly, we can draw conclusions regarding how spherical the planet is by comparing the results of GRAFT and CAP models.
3. In the case of a planet that has a random shape, and not close enough to spherical symmetry, the CAP formula is not applicable. In such case, only GRAFT algorithm can provide the desired results. This is because we can simply use different radius measurement for each session.

Additionally, actuators might be deployed underground, or above the surface of the planet, as in the case of satellites. In these cases, the effective radius used in GRAFT model changes, and the resultant net force will be different. GRAFT in such cases is applicable in a reliable manner. We believe that, for massive projects, such as deploying millions of actuators, our approach for evaluating the efficiency of the whole system may be highly needed.

## 5. Conclusions

In the scope of evaluating Internet of Things (IoT) systems, the evaluation of entities such as processors, sensors, and control devices may consider criteria such as security, latency, network load, etc. However, some IoT systems include actuator entities that are entitled to perform physical actions periodically, or upon specified data sensed by the sensors in the system. This paper proposes the GRAFT (Graph Representation using the Angle of the Force and Time) approach with the main objective to evaluate such systems in terms of force production. To show the applicability of the proposed GRAFT model, a case study was suggested, and then the model was applied on it. After that, the calculations were performed using our proposed algorithm, followed by a step-by-step explanation of this algorithm. When compared to the 100% optimal net force that can be obtained, if the Earth were not moving, our work showed that the ratio of net produced force maps to a number between 0 and 1. This was considered a problem that can be overcome by the “Restoration Value”, the concept of which is also proposed, resulting a more force-efficient system in terms of GRAFT. Although the GRAFT model was studied on the planet Earth, we believe that it can be modified to be generalized on other similar-to-Earth planets. To validate our proposed methodology, we implemented the GRAFT algorithm using the Clock-Angle-Problem formula, and compared the results of our proposed algorithm to the results obtained using our specially designed implementation. The measurements of sessions’ directions were very close, with average absolute difference of 0.155°, which indicates the validity of the GRAFT algorithm. The difference between the compared results appeared due to the fact that the Earth is not a prefect sphere, a factor that was considered in the GRAFT algorithm. 

## Figures and Tables

**Table 1:** Parameters used in the GRAFT model and their representative variables.

Parameter	Value	Notation
Rotational speed of Earth	Given	ϑ
Radius of Earth	Given	*ℜ*
Number of Sessions per day	Input by user	S
Number of Cycles in session ‘p’	Input by user	*R*[p]
Start and End time of a session	Input by user	-
Session Time	Calculated by GRAFT Algorithm	Y
Weight of the Edge	Calculated by GRAFT Algorithm	*W*
Total number of cycles in a day	Calculated by GRAFT Algorithm	*T*
Force Produced by ‘R’	Calculated by GRAFT Algorithm	A(R)
Force Produced by ‘T’	Calculated by GRAFT Algorithm	A(T)
Traveled Distance	Calculated by GRAFT Algorithm	δ
Angle of The Session	Calculated by GRAFT Algorithm	Θ
Direction of The Session	Calculated by GRAFT Algorithm	Λ

**Table 2:** The weight of each edge, which equals the ratio of the number of task cycles to the total daily number of cycles.

Session ‘*p*’	R[p]	*R*:*T*	Y[p]	W[p]
First	3	3:17	90 m	0.1765
Second	4	4:17	90 m	0.2353
Third	2	2:17	70 m	0.1176
Fourth	4	4:17	191 m	0.2353
Fifth	4	4:17	120 m	0.2353
**Total**	17	17:17	561 m	1.0000

**Table 3:** Computations results of the five tasks.

Session ‘*p*’	‘ϑ’ (m/s)	‘Y’ (min. and sec.)	‘δ’ (m)	‘*ℜ*’(m)	‘Θ’ °	(Θ/2)	‘Λ’ °
First	465.1	90 m = 5400 s	2,511,540 m	6,371,000	22.6	11.3	281.3
Second	465.1	90 m = 5400 s	2,511,540 m	6,371,000	22.6	11.3	303.9
Third	465.1	70 m = 4200 s	1,953,420 m	6,371,000	17.6	8.8	81.2
Fourth	465.1	191 m = 11460 s	5,330,046 m	6,371,000	47.9	23.95	203.95
Fifth	465.1	120 m = 7200 s	3,348,720 m	6,371,000	30.1	15.05	242.95

**Table 4:** The vertical and horizontal components of each session’s edge, calculated using the classical vectors analysis.

Task Number	Direction (Λ)	Weight (*W*)	Vertical Component	Horizontal Component
First	281.3	0.1765	−0.173	0.0346
Second	303.9	0.2353	−0.1953	0.1312
Third	81.2	0.1176	0.1162	0.018
Fourth	203.95	0.2353	−0.0955	−0.215
Fifth	242.95	0.2353	−0.209	−0.107
**Total**	-	1.0000	−0.5566	−0.1382
